# Flexible NiRu Systems for CO_2_ Methanation: From Efficient Catalysts to Advanced Dual-Function Materials

**DOI:** 10.3390/nano13030506

**Published:** 2023-01-27

**Authors:** Loukia-Pantzechroula Merkouri, Juan Luis Martín-Espejo, Luis Francisco Bobadilla, José Antonio Odriozola, Melis Seher Duyar, Tomas Ramirez Reina

**Affiliations:** 1 School of Chemistry and Chemical Engineering, University of Surrey, Guildford GU2 7XH, UK; 2 Department of Inorganic Chemistry and Materials Sciences Institute, University of Seville-CSIC, 41092 Seville, Spain

**Keywords:** CO_2_ methanation, NiRu bimetallic catalyst, dual-function material, synthetic natural gas, CO_2_ capture and utilisation, time-resolved operando DRIFTS-MS

## Abstract

CO_2_ emissions in the atmosphere have been increasing rapidly in recent years, causing global warming. CO_2_ methanation reaction is deemed to be a way to combat these emissions by converting CO_2_ into synthetic natural gas, i.e., CH_4_. NiRu/CeAl and NiRu/CeZr both demonstrated favourable activity for CO_2_ methanation, with NiRu/CeAl approaching equilibrium conversion at 350 °C with 100% CH_4_ selectivity. Its stability under high space velocity (400 L·g^−1^·h^−1^) was also commendable. By adding an adsorbent, potassium, the CO_2_ adsorption capability of NiRu/CeAl was boosted, allowing it to function as a dual-function material (DFM) for integrated CO_2_ capture and utilisation, producing 0.264 mol of CH_4_/kg of sample from captured CO_2_. Furthermore, time-resolved operando DRIFTS-MS measurements were performed to gain insights into the process mechanism. The obtained results demonstrate that CO_2_ was captured on basic sites and was also dissociated on metallic sites in such a way that during the reduction step, methane was produced by two different pathways. This study reveals that by adding an adsorbent to the formulation of an effective NiRu methanation catalyst, advanced dual-function materials can be designed.

## 1. Introduction

The reduction in carbon dioxide (CO_2_) emissions is considered the way forward so to decrease the impact of greenhouse gases on climate and thus combat global warming. The amount of CO_2_ in the atmosphere has reached 421 ppm, which is 50% higher than preindustrial levels [1]. The use of renewable energy sources to create carbon-free electricity is an effective method of dealing with CO_2_ emissions from power generation. Electric power generated by renewable sources, such as wind and solar, is variable and fluctuating; therefore, long-term storage solutions are sought [2,3,4].

The use of power-to-gas (PtG) schemes can be a solution to this issue because PtG can make use of excess electricity from renewable sources to electrolyse water and produce hydrogen (H_2_) [4,5,6,7]. Methane (CH_4_) is generally considered a way to store the surplus H_2_, owing to the safety issues of H_2_ during handling and transportation [8]. CH_4_ can be produced when CO_2_ and H_2_ react during CO_2_ methanation or Sabatier reaction (Equation (1)). The CO_2_ used in this reaction can be obtained by industrial flue gases when carbon capture is implemented; therefore, the CH_4_ or synthetic natural gas (SNG) resulting from the PtG scheme may be carbon-negative [4,8,9,10,11,12,13].

(1) $${\mathrm{CO}}_{2}+4H_{2}\to {\mathrm{CH}}_{4}+2H_{2}{O\;\Delta H}_{298K}^{o}=-164\frac{\mathrm{kJ}}{\mathrm{mol}}$$

SNG is a promising alternative fuel because it can be easily incorporated into existing industrial and transport infrastructure. The Sabatier reaction attracted a lot of attention during the 1970s oil crisis and still does due to the strict environmental regulations set by the Paris Agreement and the Glasgow Climate Pact, as well as the current energy crisis [14,15,16,17]. Several PtG demonstration plants have been built in Europe, America, and Asia to date [17,18], paving the way for more efforts towards carbon neutrality.

To date, various catalysts have been tested in the CO_2_ methanation reaction, including, Ni, Co, Ru, Rh, and Pd [11,12,13,19,20,21,22]. Ni-based catalysts are particularly attractive for commercial applications due to their low cost (EUR ~0.013/g) and good performance, as they show satisfactory CH_4_ selectivity [13,17]. Ru is the cheapest noble metal (EUR 17.9/g on 7 November 2022 [23]), it is the most active methanation catalyst, and has a much higher mass activity than Ni; however, as a precious metal, its cost can be prohibitive in most applications [8,17]. Bimetallic Ni and Ru catalysts are appealing because of their enhanced activity and stability, their increased Ni dispersion compared to monometallic Ni catalysts, and their lower cost compared to monometallic Ru catalysts [24,25,26,27,28,29,30].

Apart from the active metals, the support of the catalyst plays a vital role in its performance because the interactions between the support and the active metals alter the physiochemical properties of the catalysts. The choice of support is essential for fine-tuning the catalysts’ activity, selectivity, and lifespan. Cerium oxide (CeO_2_) is well known for its redox properties, mainly because of its unique combination of an elevated oxygen transport capacity and its ease of transformation between its reduced and oxidised states, i.e., Ce^3+^ to Ce^4+^ [31,32]. The addition of Ce to a Ni and Al_2_O_3_-based catalyst decreases its reduction temperature, enhances its stability, and prevents the formation of coke [33,34,35]. The incorporation of Zr into Ce, forming Ce_x_Zr_1-x_O_2_, results in enhanced thermal stability, astonishing oxygen capacity, and improved Ni dispersion [31,36,37,38].

By adding an adsorbent component to a methanation catalyst, a dual-functional material (DFM) can be synthesised. The concept of DFMs is an emerging area of interest for achieving integrated CO_2_ capture and utilisation [39]. Essentially, by combining an adsorbent with a catalyst, the result is a single material with the ability to both capture and convert the CO_2_ into various value-added products. As regards the process design of DFMs at a point source of emissions, at least two reactors should run in parallel, one carrying out the CO_2_ capture step and the other the CO_2_ methanation step, in order to continuously capture CO_2_. With this set up, the high exothermicity of the methanation reaction, which can generate heat management issues in a conventional reactor, is advantageous because it can supply the required heat for CO_2_ desorption and fully regenerate the adsorbent [40,41,42,43,44]. 

The most studied adsorbent in DFMs is calcium oxide due its low cost, high availability from the calcination of limestone, and its high theoretical CO_2_ uptake [40]. Other adsorbents studied for DFM technology include sodium, potassium, magnesium, cerium, and zeolites [39,40,41,42,43,44]. In terms of the active metals used, Ru has been clearly studied the most due to its ease of transformation from its oxidised species into its reduced form at low temperatures, i.e., ca. 150 °C. However, Ni has started attracting more attention due to its much lower cost compared to Ru [40,41,42].

Operando diffuse reflectance infrared Fourier transform spectroscopy (DRIFTS) is an important technique for investigating the evolution of adsorbed, desorbed, and intermediate species over a catalyst surface under working conditions by also monitoring the outlet gases. Therefore, valuable information about the reaction mechanism is obtained, and structure–activity relations can be established. Because integrated CO_2_ capture and reduction with DFMs is a new concept, understanding the reaction mechanism can help to synthesise more efficient materials in the future. Few DRIFTS studies have been conducted to date for DFMs [45,46,47,48,49,50], and it is clear that more studies are needed to further clarify the structure–activity relations.

Although several DFMs have been developed for the methanation reaction in recent years, fundamental understanding of the rate-limiting step during operation in cyclic CO_2_ capture and hydrogenation mode, as well as the connections between structure and reactivity, remains largely unknown for many such systems. Therefore, the focus of the present study is the kinetics and mechanism of NiRu-based methanation DFMs, complementing our recent work [26,51]. Herein, we employ kinetic analyses and DRIFTS to gain insights into promoter effects and the mechanism of adsorption and subsequent methanation on NiRu/CeAl and NiRu/CeZr catalysts, as well as NiRuK/CeAl DFM.

## 2. Materials and Methods

### 2.1. Catalysts Synthesis

All the materials used in this study were prepared by impregnation. The precursors used were Ni(NO_3_)_2_⋅6H_2_O (Acros Organics, Geel, Belgium) and Ru(NO)(NO_3_)_3_ solution (1.5 *w*/*v* Ru, Alfa Aesar, Ward Hill, Massachusetts, United States) for the NiRu catalytic phase and KNO_3_ (Sigma Aldrich, St. Louis, Missouri, United States) for the adsorbent. Ce_0.5_Zr_0.5_O_2_ (henceforth referred to as “CeZr”) was procured from Daiichi Kigenso Kagaku Kogyo Co., Osaka, Japan, and CeO_2_-Al_2_O_3_ (“CeAl”) was acquired from SCFa-160 Ce20 Puralox, Sasol, Johannesburg, South Africa. The detailed preparation of the catalysts [26] and dual-function material [51] is described in prior publications.

In brief, the required amounts of Ni and Ru precursors and a Ce_0.5_Zr_0.5_O_2_ or CeO_2_-Al_2_O_3_ (20–80 wt.%) support were dissolved in excess deionised water and mixed at room temperature. The excess water was then removed in a rotary evaporator under reduced pressure. Then, the catalysts were dried in air and calcined at 500 °C for 3 h (5 °C/min). In addition, the K precursor was mixed with the CeO_2_-Al_2_O_3_ support in excess deionised water, which was removed in a rotary evaporator under reduced pressure. The resulting suspension was dried and calcined at 400 °C (5 °C min^−1^). Then, the supported adsorbent, K/CeO_2_-Al_2_O_3_, was mixed with the Ni and Ru precursors in deionised water, which was then removed in the rotary evaporator. The suspension was then dried and calcined at 500 °C for 3 h (5 °C min^−1^). All the samples had 15 wt.% Ni and 1 wt.% Ru, and the DFM had 10 wt.% K_2_O. The resulting samples were named NiRu/CeZr, NiRu/CeAl, and NiRuK/CeAl.

### 2.2. Material Characterisation

#### 2.2.1. CO_2_ Temperature-Programmed Desorption

CO_2_ temperature-programmed desorption (CO_2_-TPD) was performed on the fresh NiRu/CeAl and NiRu/CeZr samples, with 50 mg of sample used in each experiment. Initially, the samples were heated to 800 °C in 10% H_2_/N_2_ (50 mL min^−1^ total flow rate) using a ramp rate of 10°C min^−1^ and held at 800 °C for 1 h in order to reduce the catalysts. Subsequently, the temperature was lowered to 40 °C in 50 mL min^−1^ pure N_2_, and CO_2_ adsorption was performed with a 10% CO_2_/N_2_ mixture for 45 min at 40 °C (50 mL min^−1^ total flow rate). Then, a N_2_ purge (50 mL min^−1^) was performed for 30 min, and after that, the temperature was increased to 800 °C (10 °C min^−1^) while maintaining 50 mL min^−1^ N_2_ flow. The data were logged using the Quadera software package, and the mass-to-charge ratio (*m/z*) of 44 corresponding to CO_2_ was monitored using an online mass spectrometer (Omni-Star GSD 320) during the temperature ramp.

#### 2.2.2. CO_2_ Capture Experiment

The CO_2_ capture capacity of the DFM was measured during a thermogravimetric analysis (TGA) experiment. The apparatus used was an SDT650 from TA Instruments. The NiRuK/CeAl sample was reduced ex situ at 800 °C for 1 h with 10% H_2_/N_2_ and a 50 mL min^−1^ total flow rate. In each run, approximately 15 mg of sample was used. The temperature was increased to 150 °C and maintained for 20 min to desorb any weakly adsorbed molecules. Then, the temperature was increased to 950 °C at a rate of 10 °C min^−1^. In the first experiment, 100 mL min^−1^ of Ar flow was used during the ramp stage, and in the second experiment, 20 mL min^−1^ of CO_2_ and 100 mL min^−1^ of Ar were used.

### 2.3. Continuous Flow Experiments

#### 2.3.1. Reactor Setup

A tubular fixed-bed quartz reactor (0.4 in ID) was used in all the experiments carried out in this study. The reactor was placed vertically in a furnace, and the catalysts were supported by quartz wool. All the experiments were conducted at atmospheric pressure, and the temperature was monitored by a thermocouple. The outlet gases, CO_2_, CH_4_, CO, and H_2_, were monitored by infrared and thermal conductivity detectors on an ABB AO2020 online gas analyser. A flow meter was employed so as to measure the outlet total volumetric flow rate of the gases. A water condenser was placed downstream of the reactor to collect all the water formed during the experiments. All the pipelines were well insulated to ensure that water did not condense before reaching the condenser.

#### 2.3.2. Continuous CO_2_ Methanation Experiment

In this work, the activity of NiRu/CeZr and NiRu/CeAl was tested in the CO_2_ methanation reaction. In each test, 0.125 g of sample was reduced at 850 °C for 1 h under a 10% H_2_/N_2_ flow at a rate of 50 mL min^−1^. The catalytic activity was measured every 50 °C in the temperature range of 500 to 200 °C. The reactant inlet ratio of CO_2_/H_2_/N_2_ was 1:4:5, with the weight hourly space velocity (WHSV) set to 24 L·g^−1^·h^−1^. The total outlet flow rate was measured at every temperature of the activity experiment with a flow meter, which was located after the reactor in sequence. This flow meter was used to allow for accurate measurements of the total volumetric flow rate and to take into account the change in the volume of the reaction mixture. The CO_2_ conversion error was ±5%, and each sample was tested twice. Equilibrium conversions were obtained via Chemstations ChemCad using the same inlet flow rates as those used in the activity experiment. Soave–Redlich–Kwong was selected as the equation of state, and a Gibbs reactor was used.

The equations used to calculate the CO_2_ conversion, CH_4_ selectivity, and CH_4_ yield are shown below (2–4). The flow rates of the respective gases are symbolised by *F*, and the subscripts in and out correspond to the inlet and outlet streams, respectively.

(2) $${\mathrm{CO}}_{2}\;\mathrm{conversion}\;\left(\%\right)=\frac{F_{{\mathrm{CO}}_{2},\;\mathrm{in}}-F_{{\mathrm{CO}}_{2},\mathrm{out}}}{F_{{\mathrm{CO}}_{2},\;\mathrm{in}}}\times 100$$

(3) $${\mathrm{CH}}_{4}\;\mathrm{selectivity}\;\left(\%\right)=\frac{\;F_{{\mathrm{CH}}_{4},\mathrm{out}}}{F_{{\mathrm{CO}}_{2},\;\mathrm{in}}-F_{{\mathrm{CO}}_{2},\mathrm{out}}}\times 100$$

(4) $$\mathrm{CO}\;\mathrm{selectivity}\;\left(\%\right)=\frac{\;F_{\mathrm{CO},\mathrm{out}}}{F_{{\mathrm{CO}}_{2},\;\mathrm{in}}-F_{{\mathrm{CO}}_{2},\mathrm{out}}}\times 100$$

Arrhenius plots were obtained to determine the apparent activation energy (Ea) of NiRu/CeAl and NiRu/CeZr. A temperature range of 200 °C to 250 °C was used, with CO_2_ conversion ranging from 5% to 25% and the activation energy determined based on the slope of the reaction rate vs. 1/temperature graph because the slope was equal to the activation energy divided by the universal gas constant (R). In this work, the apparent reaction rate is defined as the moles of the product (CH_4_) formed per active site (Ni and Ru) and per unit of time. It was assumed that all the Ni and Ru atoms of the sample mass were active to enable approximation of the activation energies. The Arrhenius expression is presented below (Equation (5)), where *k* is the rate constant, *T* is the temperature in *K*, *A* is the pre-exponential factor, and *R* is the universal gas constant (*R* = 8.314 J mol^−1^ K^−1^). The detailed mathematical derivation can be found in Appendix A.

(5) k=A×e_EaR×T

#### 2.3.3. Long-Term Stability Test

The stability of the NiRu/CeAl catalyst in the CO_2_ methanation reaction was tested in a continuous-flow quartz tube reactor, as described earlier, for 20 h. First, the sample was reduced at 850 °C for 1 h under a 10% H_2_/N_2_ flow; then, the temperature was set to 350 °C. The total flow was 200 mL min^−1^, with 10% CO_2_, 40% H_2_ and 50% N_2_. The WHSV was 400 L·g^−1^·h^−1^, and the gas hourly space velocity (GHSV) was 12,000 h^−1^. The aforementioned equations were used to measure the performance of the catalyst, and those high values were chosen to test its durability at a low reactor volume. The total outlet flow rate was measured every 30 min with a bubble meter, with the exception of night-time hours. A trend line was created based on these measurements, allowing for calculation of various parameters.

#### 2.3.4. CO_2_ Capture and Reduction Cycle

A cycle of CO_2_ capture and methanation was performed for the NiRuK/CeAl in a similar manner, as described elsewhere [51] using 0.1 g of DFM. The initial reduction was carried out at 800 °C for 1 h under a 10% H_2_/N_2_ flow at a rate of 50 mL min^−1^. A slightly lower reduction temperature was used for the DFM compared to the two catalysts to optimise the reduction temperature and avoid sintering of the adsorbent, as it was demonstrated [51] that no reduction took place after 800 °C for the NiRuK/CeAl DFM. Subsequently, the reactor was cooled down to 350 °C under N_2_ flow, and a cycle of CO_2_ capture–N_2_ purge–CO_2_ methanation was carried out. The capture and methanation steps lasted 20 min each, and the purge step lasted 5 min. During the CO_2_ capture step, 10% CO_2_ in N_2_ with a total flow rate of 50 mL min^−1^ was used, and during the CO_2_ methanation step, 10% H_2_ in N_2_ with a total flow rate of 50 mL min^−1^ was used. The flow of N_2_ was set to 45 mL min^−1^ throughout the cycle in order to be used as an internal standard to calculate the flow rates of the remaining gases according to Equation (6). The percentages of CO_2_, CH_4_, CO, and H_2_ were recorded every 5 s by an ABB analyser. The amounts of CH_4_ produced and CO_2_ desorbed were calculated based on the area under the curve of the flow rate (mL min^−1^) vs. time (min) graph.

(6) $$F_{i}=\frac{\left[i\right]}{\left[N_{2}\right]}\times F_{N_{2}}$$

### 2.4. Time-Resolved Operando DRIFTS MS Experiment

Time-resolved operando DRIFTS-MS measurements were carried out in a high-temperature reaction cell supported in a Praying Mantis (Harrick) optical system with ZnSe windows. A Thermo Nicolet iS50 FTIR spectrometer equipped with a liquid-N_2_-cooled MCT detector was used to record the spectra at 4 cm^−1^ resolution with an average of 128 scans. The outlet gases passed through a mass spectrometer (Prisma plus from Pfeiffer Vacuum), and the data were logged with Quadera software. Only the NiRuK/CeAl sample was tested. In this experiment, approximately 50 mg of sample was loaded to the cell and reduced at 600 °C for 1 h (10 °C min^−1^) with a 10% H_2_/Ar flow at a rate of 50 mL min^−1^. This reduction temperature was chosen to allow for better signal quality because higher temperatures lead to troubles with background noise. Then, the temperature was decreased to 250 °C with the same gas mixture, and 5 successive cycles of capture and reduction were performed, maintaining each step for 10 min. CO_2_ capture was performed with 10% CO_2_/Ar and reduction with 10% H_2_/Ar. The total flow rate was 50 mL min^−1^ in both cases. That temperature was chosen to better observe the intermediates.

## 3. Results and Discussion

### 3.1. Promoter Effects in the Continuous Methanation of CO_2_ over NiRu Catalysts

**Figure 1:**
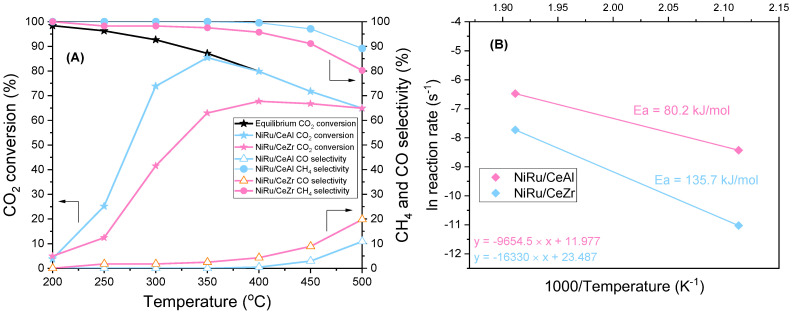
(**A**) Activity experiment of NiRu/CeAl and NiRu/CeZr showing CO_2_ conversion, as well as CO and CH_4_ selectivity and equilibrium (CO_2_/H_2_/N_2_: 1/4/5, P = 1 atm, WHSV = 24 L·g^−1^·h^−1^. (**B**) Arrhenius plots of NiRu/CeAl and NiRu/CeZr for CO_2_ methanation (*T* = 200–250 °C).

The NiRu/CeAl and NiRu/CeZr samples were tested under CO_2_ methanation conditions after they had been reduced in situ. The results of the activity experiment are presented in Figure 1A. As CO_2_ methanation is a highly exothermic reaction, the increase in the reaction temperature decreases CO_2_ conversion [29,52]. It was observed that at low temperatures, i.e., at 200 °C, the conversion was less than 5%. CO_2_ conversion reached a maximum value of 85% at 350 °C for the NiRu/CeAl and 68% at 400 °C for the NiRu/CeZr. Notably, NiRu/CeAl approached equilibrium conversion at 350 °C, showing superior activity to NiRu/CeZr.

Moreover, it was observed that the NiRu/CeAl exhibited better CH_4_ selectivity of 100% up to 350 °C. On the other hand, the NiRu/CeZr had lower selectivity than NiRu/CeAl, reaching 98% in the 250–350 °C temperature range. As temperature increased, selectivity towards CO through the reverse water-gas shift reaction (RWGS) (Equation (7)) was enhanced because RWGS was favoured at a higher temperature due to its endothermic nature. CO_2_ methanation and RWGS are competitive reactions, and careful catalyst design is needed for the desired reaction, in this case, the CO_2_ methanation reaction [22].

(7) $${\mathrm{CO}}_{2}+H_{2}\to \mathrm{CO}+H_{2}{O\;\Delta H}_{298K}^{o}=41.2\frac{\mathrm{kJ}}{\mathrm{mol}}$$

Because the conversion of CO_2_ into CH_4_ is currently the subject of considerable investigation, Table 1 summarises the performance results of Ni- and Ru-based catalysts reported in the literature. In this study, the NiRu/CeAl catalyst achieved excellent performance in the CO_2_ methanation reaction compared to similar catalysts, proving the advancement offered by this work for this reaction.

As previously mentioned, CO_2_ methanation has a kinetic regime at low temperatures (*T* < 300°C), and the CO_2_ conversions are well below the equilibrium. Therefore, the apparent activation energy calculation based on product formation can be made in a differential reactor from the Arrhenius plots. The Arrhenius plots of the two catalysts in the 200–250 °C temperature range are presented in Figure 1B, with the apparent activation energies of NiRu/CeAl and NiRu/CeZr calculated to be 80.2 and 135.7 kJ mol^−1^, respectively. It should be mentioned that these results were estimated based on two points because differential conditions were not achieved at higher temperatures. However, these estimations are in accordance with other literature data indicating that they are within the expected range. The Ea of NiRu/CeAl was comparable to previous literature findings of Ru- and NiRu-based catalysts estimated to be approximately 70 kJ mol^−1^ [8,20,29,53]. However, the Ea of NiRu/CeZr was more than 70% higher than that of NiRu/CeAl, similar to Ni-based catalysts reported in the literature [22,54,55,56]. Therefore, it was demonstrated that there was a higher kinetic barrier for the NiRu/CeZr sample compared to that of the NiRu/CeAl sample. Nevertheless, the results showcase the superiority of the latter in the CO_2_ methanation reaction.

**Table 1:** Comparison of catalytic performance of Ni- and Ru-based catalysts at 350 °C in the CO_2_ methanation reaction as reported in the literature.

Catalyst	CO_2_ Conversion (%)	CH_4_ Selectivity (%)	Ref.
15%Ni 1%Ru/CeO_2_-Al_2_O_3_	85	100	This work
15%Ni 1%Ru/Ce_0.5_Zr_0.5_O_2_	63	98	This work
4% Ru/Al_2_O_3_	80	99	[55]
12% Ni/Al_2_O_3_	55	98	[55]
15% CeO_2_ 15%Ni/Al_2_O_3_	69	97	[57]
15%Ni 2%CeO_2_/Al_2_O_3_	85	100	[33]
5% Ni/Ce_0.5_Zr_0.5_O_2_	80	99	[37]
2% Ru/30% CeO_2_/Al_2_O_3_	82	100	[58]
15%Ni/Ce_0.5_Zr_0.5_O_2_	25	86	[25]
1%Ru/15%Ni/Ce_0.5_Zr_0.5_O_2_	53	93	[25]

### 3.2. Long-Term Stability Test

Due to its higher activity, NiRu/CeAl was chosen to perform a long-term stability experiment. The reaction conditions were selected to be far from equilibrium conditions in order to better understand the catalytic behaviour. Moreover, good catalytic performance at high space velocities is favoured in industry because it is associated with a reduction in reactor volume and therefore lower capital cost.

**Figure 2:**
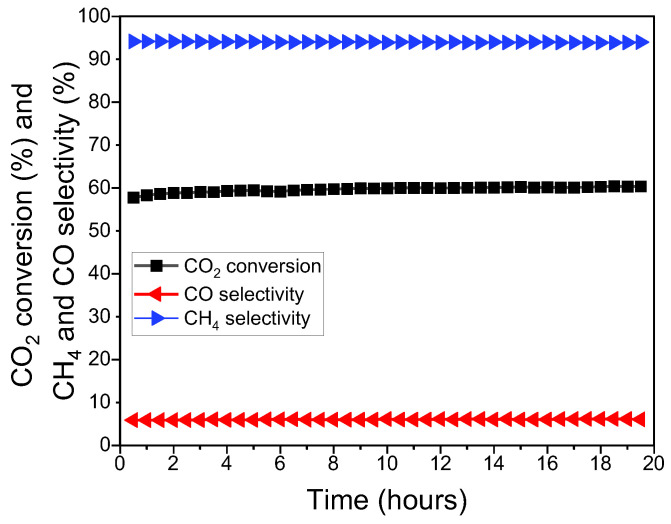
Stability experiment of NiRu/CeAl, showing CO_2_ conversion and CO and CH_4_ selectivity (CO_2_/H_2_/N_2_: 1/4/5, *T* = 350 °C, P = 1 atm, WHSV = 400 L·g^−1^·h^−1^).

The results of the stability experiment are shown in Figure 2. It was observed that the CO_2_ conversion remained stable at 60% during the 20 h experiment, indicating a great stability at such a high space velocity. The CH_4_ selectivity was 94% throughout the experiment without experiencing any drop. Accordingly, the CO selectivity was stable at 6%, and the carbon balance remained closed (100 ± 4%) during the 20 h experiment. It was therefore shown that the NiRu/CeAl was active and highly durable in the CO_2_ methanation reaction.

### 3.3. Promoter Effects on NiRu Catalysts for CO_2_ Methanation: The Effect of Surface Basicity

In general, CO_2_-TPD profiles are employed to assess the basicity of the materials and the CO_2_ adsorption sites. Depending on the temperature at which CO_2_ is desorbed, basic sites can be categorised as weak, medium, or strong. CO_2_ is desorbed from weak basic sites up to 250 °C, from medium basic sites between 250 °C and 700 °C, and from strong basic sites over 700 °C [51,56,59]. Because the typical methanation temperature is below 400 °C, the basic sites that are aimed for in this reaction are the weak and intermediate ones because the strong sites are not expected to have high reactivity. Normally, the weak peaks correspond to the CO_2_ bonded onto surface OH− groups, the medium peaks represent bidentate carbonates, and the strong peaks correspond to unidentate carbonates and strong basic surface O_2_− anions [55,60,61].

**Figure 3:**
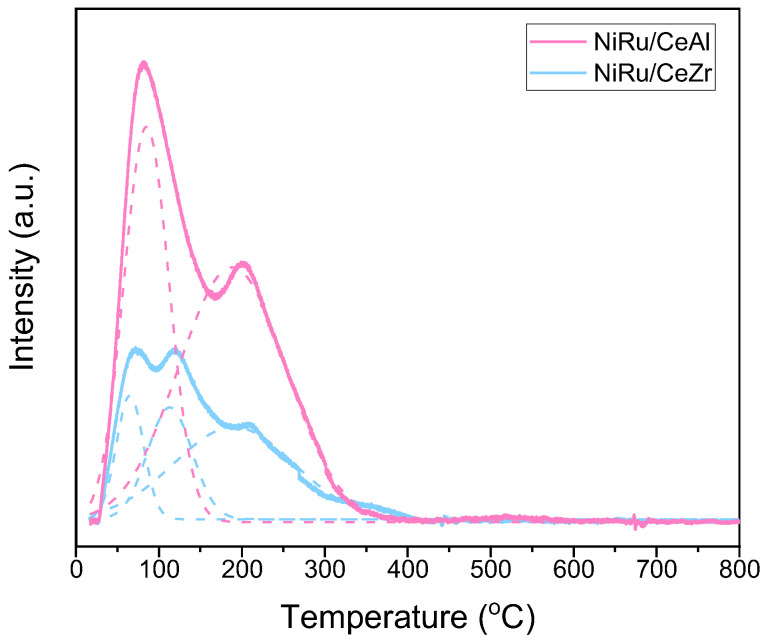
CO_2_-TPD profiles of NiRu/CeAl and NiRu/CeZr.

Figure 3 shows the CO_2_-TPD profiles of NiRu/CeAl and NiRu/CeZr. Both samples presented weak–intermediate basic sites. The first peak of both samples at ca. 80 °C and the second of peak the NiRu/CeZr at 120 °C correspond to CO_2_ desorption from the weak Bronsted OH− groups. The peaks located at ca. 200 °C were assigned to the decomposition of bidentate carbonates formed on oxygen–metal pairs [55,60,61]. In addition, Figure 3 implies that there were more weak basic sites on the NiRu/CeAl sample based on the signal intensity. Because the basic sites that participated in this reaction were the weak–intermediate sites, it was concluded that the NiRu/CeAl sample was able to provide more sites for CO_2_ adsorption, leading to better catalytic activity. Therefore, its increased performance was attributed to its enhanced basicity, as reflected by the CO_2_-TPD results, in addition to its lower activation energy barrier.

In addition to the CO_2_-TPD results presented herein, Table 2 summarises some important characterisation information about the two catalysts and the DFM obtained in our previous studies [26,40]. It can be concluded that besides the enhanced basicity and lower kinetic barrier of the NiRu/CeAl catalyst compared to the NiRu/CeZr catalyst, its higher surface area, better Ni dispersion, and smaller particle size were also crucial aspects that led to better catalytic activity.

### 3.4. Adding CO_2_ Capture Functionality to Synthesise a Methanation DFM

Because NiRu/CeAl demonstrated good activity and stability in the CO_2_ methanation reaction, it was selected to be upgraded to a dual-function material by incorporating an adsorbent into its catalytic formulation. In this work, the chosen adsorbent was potassium. The aim of the TGA experiment was to observe the capturing ability of the reduced K-based DFM and, consequently, its best-performing temperature by simply ramping the temperature with CO_2_ and Ar. An experiment with only Ar was also carried out on the reduced DFM to be used as reference for any material degradation and any weakly desorbed species.

**Figure 4:**
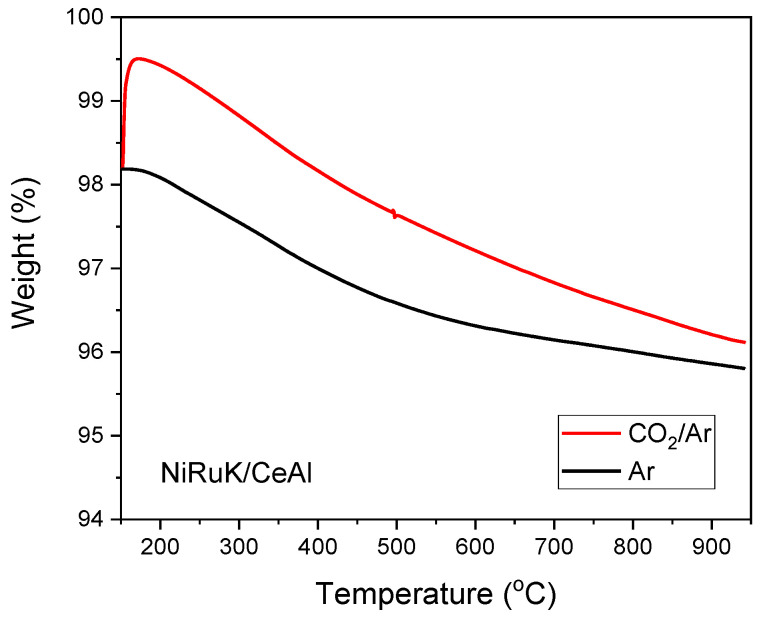
Weight change of NiRuK/CeAl with increasing temperature under Ar and CO_2_/Ar flows.

The results of this study are displayed in Figure 4. It was observed that the DFM was able to adsorb CO_2_ immediately once it came into contact with the CO_2_-containing stream at 150 °C, with a maximum weight uptake slightly above that temperature, i.e., at 170 °C. At that temperature, the DFM adsorbed 0.013 mg of CO_2_/mg of sample or, alternatively, 0.3 mmol of CO_2_/g of sample. Moreover, it appeared that the DFM was able to keep that amount of CO_2_ adsorbed onto its surface up to 400–500 °C. However, when the temperature was increased beyond that temperature range, a decline in the weight percentage was observed, indicating that the formed carbonates were not strong enough and that their decomposition started to take place, yielding K_2_O and CO_2_ [62]. At the end of the experiment (950 °C), only a very small amount of CO_2_ remained on the surface: 0.0003 mg of CO_2_/mg of sample or 2.4% of the initial CO_2_ uptake, which may also be attributed to experimental error.

These results are in accordance with previous literature findings, which showed that K-based adsorbents are appropriate for low–intermediate temperature applications when *T* < 400 °C [42,51,63,64,65,66,67]. Therefore, the use of K-based DFMs in the CO_2_ methanation reaction, which also occurs at low–intermediate temperatures, is desirable. It is worth noting that the current adsorption results should be compared with the results for other DFMs only, despite the potential existence of better CO_2_ adsorbents, because it is important to understand their adsorption behaviour under dynamic DFM operation even if the experiments are conducted under ideal conditions.

### 3.5. CO_2_ Capture and Reduction Study over NiRuK/CeAl DFM

As demonstrated by the capture experiment in TGA, the NiRuK/CeAl sample was able to capture CO_2_ in the typical methanation temperature range. In addition, the NiRu/CeAl catalyst showed impressive activity, selectivity, and stability in the methanation reaction, which motivated the development of a DFM using this catalyst. A cycle of CO_2_ capture, N_2_ purge, and CO_2_ methanation was performed in order to demonstrate the ability to ‘transform’ a typical methanation catalyst into a highly effective DFM. The temperature chosen for this experiment was 350 °C because at this temperature, adsorption was feasible according to the CO_2_ capture experiment in TGA, and the NiRu/CeAl catalyst exhibited the highest conversion according to the activity experiment. Moreover, at this temperature, the DFM could take advantage of the reversibility of the captured CO_2_ so as to transform it into CH_4_ more easily. The DFM mode of operation differs from typical adsorption processes, which require a change in temperature or pressure for the desorption step to occur. In particular, the fact that DFMs can operate under atmospheric pressure, thus avoiding pressure swing adsorption (PSA), is one of their main assets, leading to lower costs. It is worth noting that in comparison with the continuous CO_2_ methanation experiments reported in previous sections, these cyclic experiments operate under dynamic conditions. Therefore, it is not obvious how the DFM will behave in terms of methanation activity compared to steady-state operation of the parent catalyst [40].

**Figure 5:**
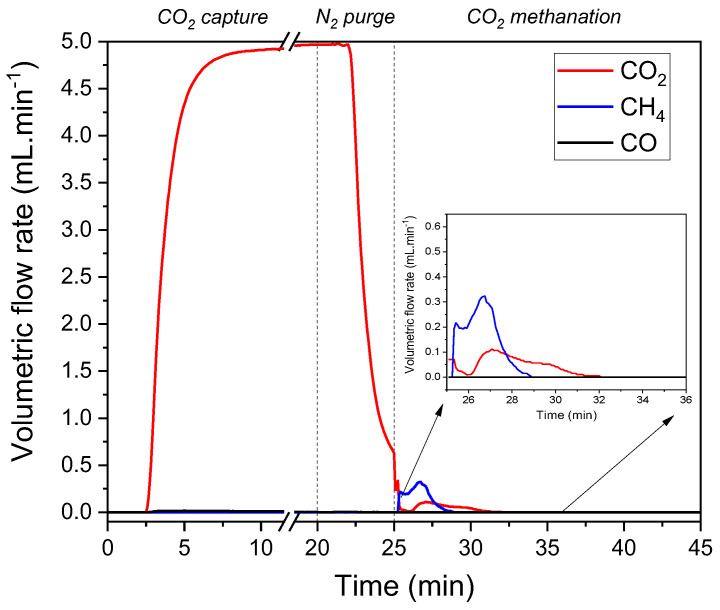
Volumetric flow rates (mL min^−1^) of CO_2_, CO, and CH_4_ during CO_2_ capture and methanation experiment with NiRuK/CeAl (*T* = 350 °C, P = 1 atm).

Figure 5 shows the results of the CO_2_ capture and methanation experiment. It was noticed that CO_2_ breakthrough occurred within the first 3 min of exposing the DFM to the CO_2_-containing stream. Breakthrough was estimated to have taken place within the first 10–15 s, but there was an anticipated time delay due to the setup of the pipelines and reactor. This material displayed the typical CO_2_ adsorption curve, as adsorption includes a fast step followed by a slower step [51,68]. During methanation, only CH_4_ and CO_2_ were detected. NiRuK/CeAl displayed 100% CH_4_ selectivity despite the fact that K is typically used as a promoter in the RWGS reaction [69,70,71,72,73]. The amounts of CH_4_ produced and CO_2_ desorbed were calculated based on the area under the curve in Figure 5. Hence, 0.264 mol of CH_4_/kg of sample was produced, and 0.139 mol of CO_2_/kg of sample was desorbed. The sum of the CH_4_ moles formed and the CO_2_ moles desorbed was higher than the moles of CO_2_ adsorbed in the TGA experiment. However, this was expected because there was still some CO_2_ in the gas phase before the methanation step started, as shown in Figure 5; moreover, the CO_2_ adsorption temperature was constant at 350 °C in this experiment but not in the TGA experiment. Nevertheless, the sum of the CH_4_ production and CO_2_ desorption capacities were consistent with similar K-based DFMs found in the literature [40,74]. In addition, a delay in CO_2_ desorption was detected, proving that the heat released from the methanation reaction supplied the required heat for CO_2_ desorption to take place. Furthermore, it should be noted that CH_4_ was detected in the first 3 min of the methanation step, indicating that the CO_2_ capture and methanation steps can have the same duration in future experiments, as opposed to the fact that material regeneration has typically been a slower step in other DFM studies [74,75].

The methanation capacity obtained in this work is comparable to previous K-based DFM results reported in the literature or slightly lower [40,51,67,74]. However, a very important parameter that needs to be taken into consideration is the N_2_ purge step. In the typical methanation temperature range, CO_2_ is weakly adsorbed onto the surface of the DFM. This means that when N_2_ purge takes place after the capture step, weakly adsorbed CO_2_ is desorbed, affecting the DFM methanation capacity and demonstrating that the desorption capacity of DFMs affects their performance, in agreement with previous studies [51]. Consequently, in the future, it is advisable to perform experiments by tracking the outlet flow rates and shortening the times of capture and methanation steps so as to continuously clean the effluents from CO_2_ and produce valuable products [62]. However, when performing O_2_-containing CO_2_ capture followed by a hydrogenation reaction, a N_2_ purge is required for safety reasons. Other uncommon impurities, such as NOx and SOx, which are present in real-world applications, also need to be taken into consideration, as they affect the DFM performance [76,77,78]. Furthermore, material recyclability allowing for the sustainable recovery of the main metals from the spent catalyst is another important aspect when designing such materials.

### 3.6. Time-Resolved Operando DRIFTS-MS Experimental Analysis

**Figure 6:**
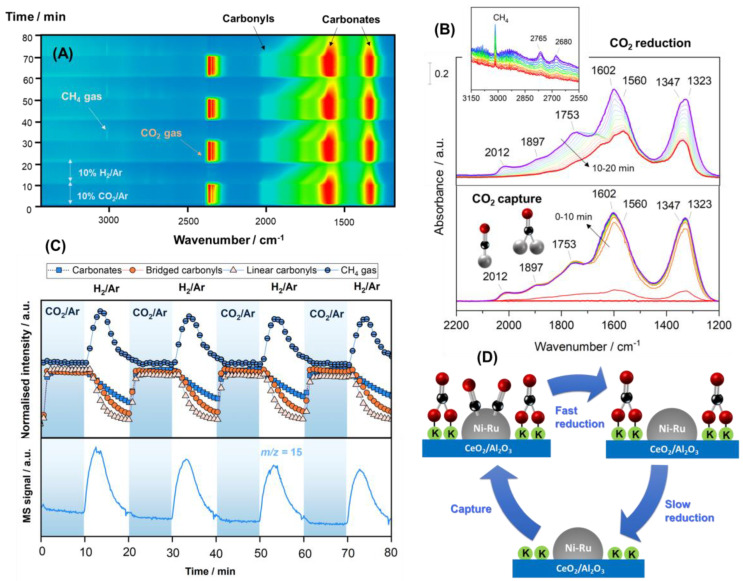
(**A**) Bidimensional representation of time-resolved DRIFTS spectra on a reduced NiRuK/CeAl sample at 250 ºC during capture/reduction cycles (10% CO_2_ in Ar and 10% H_2_ in Ar, both at 50 mL min^−1^). The spectrum after activation pretreatment was taken as background. (**B**) Representative evolution of spectra during the first cycle of capture/reduction. (**C**) Evolution of selected IR bands and of a *m/z =* 15 (CH_4_) signal during the capture/reduction cycles. (**D**) Illustrative sketch of the capture/reduction process on NiRuK/CeAl.

In order to achieve improved understanding of the surface intermediates involved in CO_2_ capture and reduction processes, we further performed an operando DRIFTS-MS study on the most optimal material, NiRuK/CeAl. For this purpose, the DFM was reduced in situ at 600 °C for 1 h in a flow of 50 mL min^−1^ of 10% H_2_/Ar. The repeated CO_2_ capture and reduction cycles were carried out by alternating a feed flow of 50 mL min^−1^ of 10% CO_2_ in Ar versus 10% H_2_ in Ar under isothermal conditions at 250 °C. Time-resolved DRIFTS spectra were successively recorded, and the dynamics evolution of the bands related to surface species was displayed in a 2D map, as shown in Figure 6A. It should be mentioned that all DRIFTS spectra were subtracted with respect to the spectrum of the surface after activation for clarification. As shown in Figure 6A, the development of bands ascribed to gaseous CO_2_ (2360 cm^−1^) and the symmetric/asymmetric stretching OCO vibrations characteristic of carbonate species were clearly visible under the CO_2_ capture feed [79]. In addition, it is worth observing the appearance of bands associated with CO adsorbed on metal sites, revealing that during the capture step, CO_2_ may be dissociated into CO* and O* adsorbed species. It is well known that ruthenium decreases the energy barrier for CO_2_ dissociation and facilitates the subsequent hydrogenation of CO* adsorbed, yielding CH_x_ fragments [80]. Likewise, it is also possible to reduce CO_2_ to CO on the oxygen-deficient sites of ceria promoter and CO to migrate to metal sites [81]. After switching from the CO_2_ capture to H_2_ reduction stream, all the bands gradually decreased, and a new band emerged at 3014 cm^−1^, which was attributed to gaseous methane.

A closer inspection of the DRIFTS spectra collected during the first cycle of capture/reduction is displayed in Figure 6B. As can be observed, the spectra recorded during the CO_2_ capture step (0–10 min) were dominated by the development of two intense bands in the 1800–1200 cm^−1^ region. These bands were ascribed to the symmetric and asymmetric stretching vibrations related to carbonate-like species with different adsorption geometry on the Al-O-K and Ce-O-K basic sites of the support. In more detail, the pair of bands at 1602–1323 cm^−1^ corresponded to bidentate carbonates (Δν_3_ splitting = 321), whereas the bands at 1560–1347 cm^−1^ (Δν_3_ splitting = 213) were associated with bridged carbonates [79,82]. It should be stressed that bands related to bicarbonate species were scarcely observed. The absence of bicarbonates can be explained by the fact that alkali promotion neutralises the hydroxyl groups on the alumina surface, which are responsible for the formation of bicarbonate species by reacting with CO_2_ [83,84]. On the other hand, another band around 1753 cm^−1^ was also notable, which was ascribed to the formation of K_2_CO_3_ over agglomerated potassium oxide particles when exposed to a CO_2_ stream [85]. With respect to the bands developed at higher frequencies in the 2100–1800 cm^−1^ region, two bands at 2012 and 1890 cm^−1^ were clearly appreciable. These features were ascribed to linear and bridged carbonyl-adsorbed species, respectively, on nickel metallic sites [86]. Presumably, the observed vibrational frequencies were shifted to relatively lower values than those reported for nickel monometallic catalysts. This fact could be attributed to the presence of ruthenium, which induced electron transfer from Ru to Ni sites, resulting in higher electron cloud density on Ni metallic sites. Moreover, the absence of Ru carbonyl bands also indicated the interaction between Ni and Ru, meaning that a synergy between the two active metals took place [24,30]. Alloy formation could be possible according to our previous work [51], but more studies are needed to confirm that. Nevertheless, the presence of carbonyl-adsorbed species reveals that CO_2_ suffered partial dissociation on metal sites or reduction on oxygen vacancies.

Next, the series of DRIFTS spectra collected during the following 10 min under 10% H_2_/Ar flow (Figure 6B) showed that the features related to metallic carbonyl species rapidly vanished with the concurrent production of gaseous methane (Figure 6B inset). It was also evident that the intensity of carbonate bands (1600–1200 cm^−1^) decreased with the exposure time, but those species still remained after 10 min of reduction. This observation clearly showed that the reduction rate of basic carbonates was much slower than that of carbonyl species. Notably, the band at 1730 cm^−1^ exhibited a shift to low frequency with the simultaneous emergence of two weak bands at 2765 and 2680 cm^−1^ in the C-H stretching region (Figure 6B inset). After 7 min of 10% H_2_/Ar exposure, all these bands decreased notably in intensity. Solymosi and Knözinger [87] observed similar features and reported that they were related to the formation of potassium formates from the reduction of potassium carbonates. It is worth mentioning that the associative mechanism of CO_2_ methanation proposes that carbon dioxide reacts with surface hydroxyls on the support, producing bicarbonates that are progressively hydrogenated to methane passing through the formation of formates at the metal–support interface [88,89]. Our findings suggest that formates may also be formed on the potassium sites and subsequently be hydrogenated toward methane, following an alternative pathway in the absence of bicarbonates.

Moreover, Figure 6C shows the evolution of the IR band intensities related to carbonates (1323 cm^−1^), bridged carbonyls (1897 cm^−1^), linear carbonyls (2012 cm^−1^), and gaseous methane (3016 cm^−1^) as a function of the time on stream during the capture/reduction cycles. Understanding the evolution trend of adsorbed *CO species and carbonates is critical to determine the reduction mechanism of the captured CO_2_. It was evident that the intensity of both types of carbonyls decayed much faster than that of carbonates such that they can be distinguished between fast and slow reduction steps, as shown in Figure 6D. The linearly bounded *CO species decreased sharply and diminished within 4–5 min after switching the stream to 10% H_2_ in an Ar feed. The bridged carbonyls were somewhat less reactive than the linear carbonyls, but they also decreased moderately fast. These observations are in line with the results reported by Vogt et al. [90], who found that linearly adsorbed CO is more weakly adsorbed than carbonyls in bridge conformation; thus, the rate-determining step in CO_2_ methanation is governed by the concentration of linearly bounded CO species and their availability to be hydrogenated into methane. Meanwhile, the intensity of the band of carbonates depleted more slowly, remaining after 10 min of reduction. Simultaneously, it was evident that methane was produced rapidly during the first minutes, and its production decayed progressively. Even if some carbonates species remained on the surface after 10 min, most were eventually converted into CH_4_, as shown in Figure 6C. Although we could not rule out the presence of inactive carbonate species, the initial rapid production of methane, along with a slight decay in production, was consistent with the coexistence of fast (carbonyl) and slow (carbonate) reduction pathways, as depicted in Figure 6D.

In summary, we can rationalise that during the capture step, CO_2_ is dissociated on metal sites or reduced in oxygen-deficient sites, producing linear and bridged carbonyl-adsorbed species. On the other hand, CO_2_ is also adsorbed on the support basic sites, forming carbonate-like species. In the reduction step, carbonyl species are rapidly hydrogenated into CH_4_, whereas carbonates are more stable and require a longer time to be reduced. This study provides a primary approach to understanding the capture/reduction process at the fundamental level so as to design more efficient dual-function materials. In addition, the operando DRIFTS-MS results reported herein demonstrate that our DFM achieved promising results in terms of material stability, and more studies are currently underway to better comprehend its behaviour over time, especially under non-realistic conditions.

## 4. Conclusions

Two catalysts, NiRu/CeAl and NiRu/CeZr, were tested in the CO_2_ methanation reaction. It was observed that the NiRu/CeAl sample was more active and selective in the CO_2_ methanation reaction compared to the NiRu/CeZr sample, as it almost reached equilibrium conversion at 350 °C with 100% CH_4_ selectivity. Furthermore, during the stability experiment of the NiRu/CeAl at a high space velocity, i.e., 400 L g^−1^ h^−1^, which lasted 20 h, no drop in CO_2_ conversion was detected.

The NiRu/CeAl catalyst was upgraded by incorporating an adsorbent, i.e., potassium, so as to obtain a dual-function material. The sample was tested in a cycle of CO_2_ capture–N_2_ purge–CO_2_ methanation, exhibiting a methanation capacity of 0.264 mol of CH_4_/kg of sample and CO_2_ desorption capacity of 0.139 mol of CO_2_/kg of sample. By carrying out time-resolved operando DRIFTS, it was demonstrated that CO_2_ was dissociated on the metallic sites and reduced on the oxygen-deficient sites of Ce. Moreover, K_2_CO_3_ and carbonate-like species were observed on the Al-O-K and Ce-O-K basic sites of the support. During CO_2_ methanation, it was demonstrated that the carbonyl and carbonate species were converted into CH_4_ at different rates.

Overall, in this work, we have investigated the effectiveness of Ni-Ru catalysts for the CO_2_ methanation reaction and performed kinetic and mechanistic analyses. Notably, the comprehension of the CO_2_ adsorption and subsequent methanation mechanisms of our dual-function material was accomplished. Further fundamental understanding is nevertheless needed to fine-tune the formulation of these materials. In any case our dual-function materials are able to both capture and convert CO_2_ into synthetic natural gas showcasing an innovative approach to address current environmental issues. 

## Figures and Tables

**Table 2:** Summary of characterisation data of NiRu/CeAl, NiRu/CeZr [26], and NiRuK/CeAl [51].

Material	Metal Loading (%)	Adsorbent Loading (%)	BET (m^2^/g)	Pore Volume (cm^3^/g)	Ni Particle Size (nm) ^1^	Crystalline Phases ^2^	H_2_-TPR Main Reduction Peaks (°C)
15%Ni 1%Ru/CeO_2_-Al_2_O_3_	15-1	-	141	0.29	12	Ni^0^, Ru^0^, Al_2_O_3_	130, 380
15%Ni 1%Ru/Ce_0.5_Zr_0.5_O_2_	15-1	-	60	0.18	34	Ni^0^, NiO, Ce_0.5_Zr_0.5_O_2_	150, 320
15%Ni 1%Ru 10%K_2_O/CeO_2_-Al_2_O_3_	15-1	10	170	0.40	10	Ni^0^, CeO_2_, Al_2_O_3_	190, 350, 460

^1^ Estimated nickel crystalline size based on Scherrer equation (2θ° = 44.48). ^2^ Crystalline phases detected in their reduced XRD profiles.

## Data Availability

Original data will be available upon request.

## Appendix A

(A1)
$$r=k\times p_{H2}^{\alpha }\times p_{\mathrm{CO}2}^{\beta }$$

(A2)
∴ r=A×e_EaR×T×pH2α×pCO2β

(A3)
$$lnr=lnA-\frac{Ea}{R\times T}+\alpha \times lnp_{H2}+\beta \times lnp_{\mathrm{CO}2}$$

The Arrhenius expression is copied from the main text (Equation (5)), where *k* is the rate constant, *T* is the temperature in *K*, *A* is the pre-exponential factor, and *R* is the universal gas constant (*R* = 8.314 J mol^−1^ K^−1^). In addition, *r* is the reaction rate, p_H2_ is the H_2_ partial pressure, *p*_CO_2__ is the CO_2_ partial pressure, and *α* and *β* the reaction orders.

In this case, the reaction orders and the partial pressures of H_2_ and CO_2_ remained the same. Therefore, a linear correlation between the *lnr* and the temperature reciprocal may be assumed, making the slope a function of the apparent activation energy and universal gas constant.
